# Elucidation of the effects of autochthonous starter on nitrogen‐containing compounds during fermentation of *Yujiangsuan* by metabolomics

**DOI:** 10.1002/fsn3.3674

**Published:** 2023-10-19

**Authors:** Shirui Yu, Wenkang Hu, Lina Ma, Yin Luo, Xuefeng Zeng, Shanjun Tian

**Affiliations:** ^1^ Department of Food Science and Engineering Moutai Institute Renhuai China; ^2^ School of Liquor and Food Engineering Guizhou University Guiyang China; ^3^ College of Agriculture Guizhou University Guiyang China

**Keywords:** GC‐TOF‐MS, metabolomics, nitrogen‐containing compounds, *Yujiangsuan*

## Abstract

To understand the role of microorganisms in nitrogen (N)‐containing compound changes during the processing of *Yujiangsuan* by autochthonous starter cultures, the GC‐TOF‐MS‐based metabolomics method was adopted to investigate the effects of *Weissella cibaria* and *Lactobacillus plantarum.* The results demonstrated that inoculation of autochthonous strains led to differential metabolites, such as fatty acids, organic oxygen compounds, and carboxylic acids on day 4 to day 12 of fermentation. The N‐containing compounds under the inoculated fermentation group showed a faster relative concentration change. Nucleotide metabolism and arginine and proline metabolism exerted an influence on the formation of N‐containing compounds. Apart from that, the effect of *W. cibaria* and *L. plantarum* on the hydrolysis of macromolecules was the main factor causing differences in major N‐containing compounds.

## Graphical abstract

The effect of the HY treatment on the hydrolysis of macromolecular substances was the main factor causing differences in major nitrogenous compounds, such as amino acids, peptides, purines, pyrimidines, and nucleosides, with different treatments. It is believed that the findings provide theoretical support for enhancing *Yujiangsuan* fermentation and producing safe products.

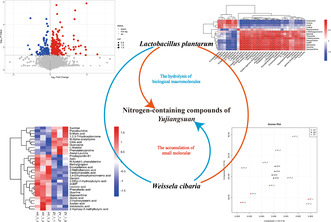

## 1 INTRODUCTION

*Yujiangsuan* (YJS), a traditional fermented fish–chili paste with local characteristics and a long history, is popular in southwest China for its unique flavor. As a mixture of red pepper, fresh fish, white wine, ginger, and salt, it contains organic acids, lipids, amino acids, and alkaloids (Hua et al., 2022), which make it taste sour, sweet, umami, and salty. Besides, YJS has a much higher concentration of free amino acids than typical fermented chili peppers (Yang et al., 2021). In addition, microbial fermentation is a key step in the formation of nutrients and unique flavor compounds of YJS (Jiang et al., 2022), and the native spontaneous strains are used as fermentation starters in the traditional production process. Actually, the unstable microbial sources in traditional processes lead to long fermentation times and challenges in quality control measures. Moreover, the contamination of undesired microbes jeopardizes the safety of fermented products (Hu et al., 2021). In this context, inoculated fermentation with the dominant microorganisms in fermented products can ensure high quality, high efficiency, and safety. Hua et al. (2020) demonstrated the positive effect of the inoculated microbial starter on the improvement of quality in YJS. The production of biogenic amines in YJS was effectively inhibited, and ester volatile flavor substances were significantly increased by inoculation with aroma‐producing yeast and bacterial starters.

Nitrogen (N)‐containing compounds play a very complex role in fermented foods; on the one hand, they do provide foods with a rich flavor. N‐containing compounds, such as amino acids and oligopeptides and derivatives, generated from protein hydrolysis (Yao et al., 2021), purines and pyrimidines derived from nucleic acid hydrolysis, and their derivatives (Zheng et al., 2020), were important sources of flavor, and the involvement of microorganisms resulted in complex changes in these substances. The acceptance of N‐containing flavors by consumers depends on their food culture. On the other hand, the degradation of N‐containing compounds during fermentation can lead to the generation of potentially toxic compounds such as nitrite, volatile salt nitrogen (TVB‐N), and other biogenic amines, which can cause considerable flavor changes and negative impacts on human health and product quality (Bekhit et al., 2021). By utilizing key fermentation starters to regulate the generation of N‐containing compounds during the fermentation process, the flavor and safety of YJS will be maintained.

*Weissela cibaria* AT22 and *Lactobacillus plantarum* 3072 were isolated and selected by Liu et al. (2020) in naturally fermented YJS. The two strains dominate during the early period of fermentation, growing and multiplying rapidly, and producing organic acids and other beneficial compounds. As additions to YJS starters, the two strains have a higher content of amino acids and TCA‐soluble peptides and obviously inhibit the production of nitrite, TVB‐N, and thiobarbituric acid (TBA) compared with the naturally fermented one.

Metabolomics is frequently employed in the field of life sciences to examine and discover changes in the metabolites of different complex systems. Non‐targeted or multivariate metabolomics analysis has been successfully performed in identifying fermentation species and determining the metabolic pathways in the field of fermented food research (Dan et al., 2022; Lai et al., 2021; Zotta et al., 2022). Beyond that, they can also be utilized to find and recognize markers, as well as to monitor and optimize the fermentation process (Chen et al., 2022; Hu et al., 2022).

In this study, a metabolomic method based on gas chromatography‐tandem time‐of‐flight mass spectrometry (GC‐TOF‐MS) was used to investigate the effects of fermentation with mixed strains of *W. cibaria* AT22 and *L. plantarum* 3072 exogenously added to YJS on the N‐containing compounds during fermentation. It is believed that the findings provide theoretical support for enhancing YJS fermentation and producing safe products.

## 2 MATERIALS AND METHODS

### 2.1 Starter culture

*Weissella cibaria* AT22 and *L. plantarum* 3072, able to enhance amino acid content and reduce toxic N‐containing compounds, were derived from traditional naturally fermented YJS. Both strains were preserved at the Department of Food Science and Engineering of the Moutai Institute, and stored at −80°C. Then, the two strains were added to the MRS liquid medium and activated at 37°C for 18 h before the test. After that, the culture was transferred to a sterile centrifuge tube, centrifuged at a speed of 2656 ×g for 1 min, and the sediment was washed with sterile normal saline, followed by the cleaned sediment being diluted to obtain a bacterial suspension with a live bacterial concentration of 107 CFU/mL as the starter.

### 2.2 Preparation of YJS

Fresh chilies and loaches, purchased in the agricultural market at Renhuai, were cleaned and chopped. The chilies were weighed and combined with 10% (w/w) loach and 4% (w/w) ginger. For the inoculation fermentation group (HY), 7% (w/w) salt, 3% (w/w) baijiu, and 1% (v/w) starter were mixed and fermented at 25°C. For the traditional fermentation method (ZY), the starter was not added as the control group. The samples collected on days 0, 4, 8, 12, and 16 of fermentation for analysis are represented, respectively, as HY1, HY2, HY3, HY4, and HY5, and ZY1, ZY2, ZY3, ZY4, and ZY5.

### 2.3 Extraction of metabolites

Samples (50 mg) were precisely weighed and transferred to an Eppendorf tube, containing 1000 μL of extract solution (3:1 methanol:water, v/v, precooled at −40°C, containing an internal standard). The samples were vortexed for 30 s, homogenized at 35 Hz for 4 min, and sonicated for 5 min in an ice‐water bath. Here, it should be noted that the homogenization and sonication steps were repeated three times. Afterwards, the sample was kept at −40°C for 1 h and then centrifuged at 4°C and 10625 ×g for 15 min. The supernatant (200 μL) was transferred to an Eppendorf tube, with 40 μL being taken from each sample and mixed into the QC samples. At last, the extract was dried in a vacuum concentrator.

Forty microliters of methoxyl amine salt solution (methoxyl amine hydrochloride dissolved in pyridine, 20 mg/mL) were added to the dry extract, mixed well, and maintained at 80°C for 30 min. Then, 60 μL BSTFA (containing 1% TMCS, v/v) was added to the sample, mixed well, and maintained at 70°C for 1.5 h. After cooling to room temperature, 5 μL FAMEs (dissolved in chloroform) were added to the mixture.

### 2.4 GC‐TOF‐MS analysis

GC‐TOF‐MS was performed using the Agilent 7890 gas chromatograph and the time‐of‐flight mass spectrometer with the DB‐5MS capillary column. The sample volume injected into the GC system was 1 μL; the carrier gas was helium; the forward sample flow rate was 3 mL/min; and the gas flow rate was 1 mL/min. The temperature program was as follows: 50°C being held for 1 min, then increasing to 310°C at 10°C/min and being kept for 8 min. The temperature of the injection port and transfer line was 280°C, and that of the ion source was 250°C. The energy in the electron impact mode was −70 eV, and the mass spectral data range was m/z 50–500. After a solvent delay of 6.25 min at a speed of 12.5 spectra per second in the full scan mode, the resulting extracts of each sample were analyzed with uninterrupted chromatography.

### 2.5 Statistical analysis

Microsoft Excel was used for data collation of trial results, and multivariate statistical analysis was made using SIMCA 16.0.2. Besides, R software (R‐4.1.3) was adopted for ANOVA, cluster analysis, correlation analysis, and graphing. Furthermore, metabolic pathway analysis was performed in the KEGG database (https://www.kegg.jp/).

## 3 RESULTS AND DISCUSSION

### 3.1 Metabolite composition analysis

**FIGURE 1:**
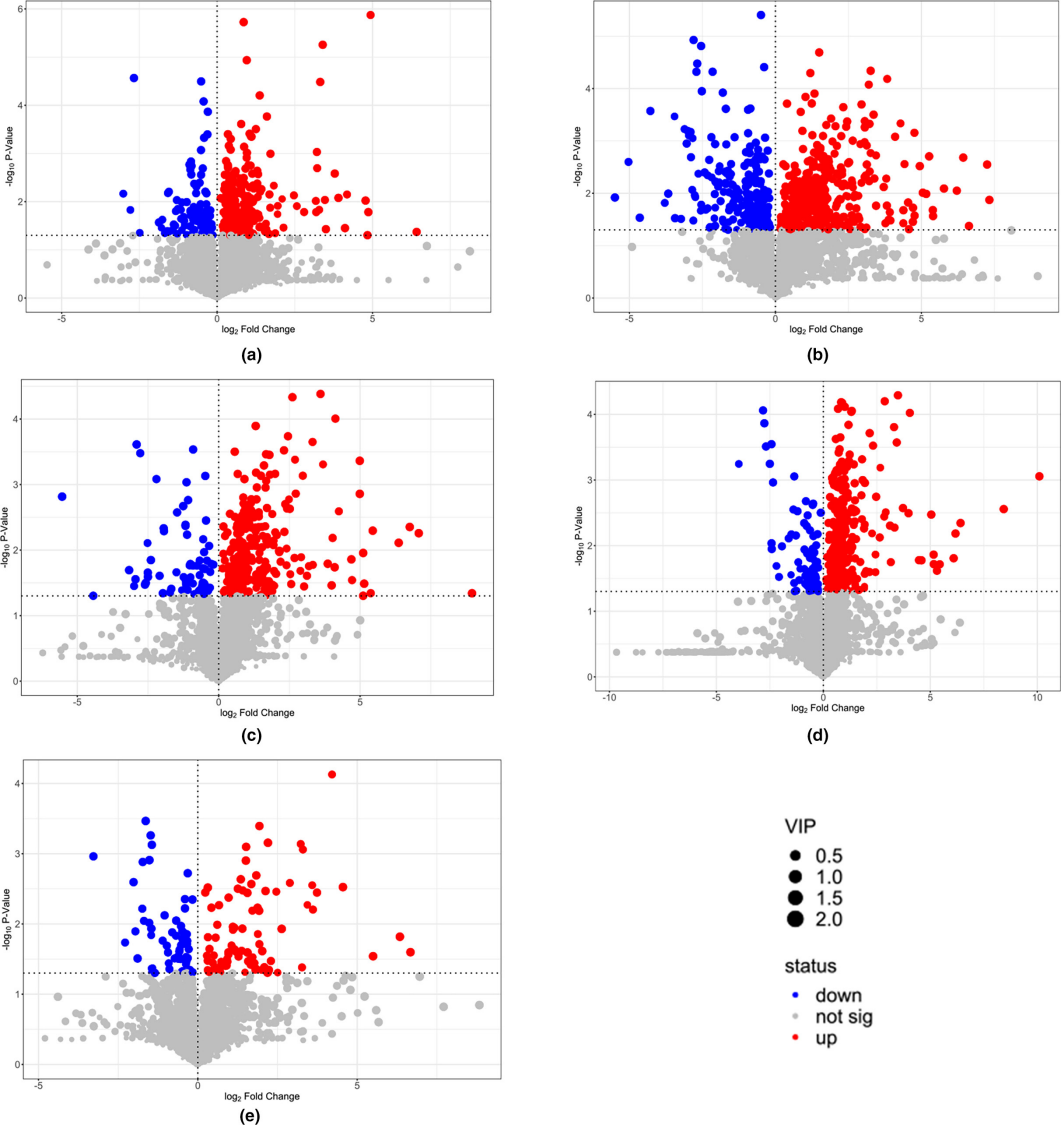
Volcano plots of differential metabolites between HY and ZY: a–e are the Volcano plots on days 0, 4, 8, 12, and 16, respectively.

In total, 3759 ion peaks from YJS samples were extracted and retained by conducting GC–MS/MS analysis. Orthogonal partial least‐squares discrimination analysis (OPLS‐DA) was modeled for the statistical analysis of trial data. Other than that, the *p*‐values, fold change, and variable importance (VIP) of the OPLS‐DA model (*p* < .05, VIP > 1) were used as thresholds for differential metabolite screening (Saccenti et al., 2014), and the results are shown as volcano plots (Figure 1). The inoculation of *W. cibaria* and *L. plantarum* resulted in obvious differences concerning YJS fermentation. Compared with ZY, HY got 242 upregulated and 104 downregulated peaks on day 0, 378 upregulated and 243 downregulated peaks on day 4, 295 upregulated and 68 downregulated peaks on day 8, 336 upregulated and 87 downregulated peaks on day 12, and 87 upregulated and 63 downregulated peaks on day 16. Moreover, the difference between HY and ZY continuously increases through fermentation, reaching the peak on day 12, and then suddenly decreases. It is suggested that, compared with traditional fermentation, the inoculation of strains would cause significant changes in substance concentrations during the early stages of YJS fermentation.

**FIGURE 2:**
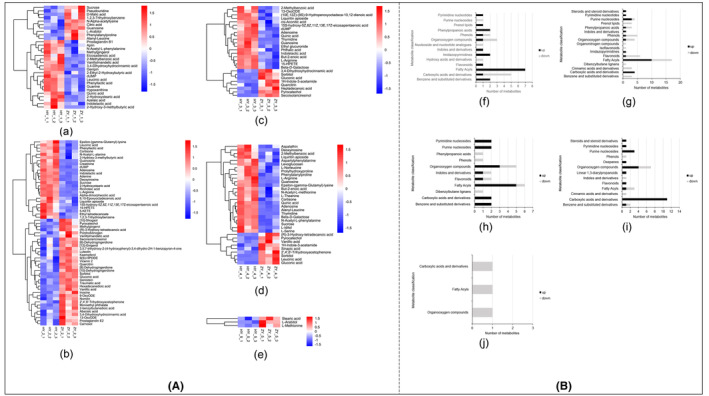
Heat map of different metabolites of YJS at different fermentation stages (A) Classification map of differential metabolites (B) (f–j) represent the classification results on days 0, 4, 8, 12, and 16, respectively.

By qualitatively matching based on secondary mass spectra, 225 metabolites were identified from ion peaks. Clustered heatmaps were used to visually describe the noticeably different metabolites (*p* < .05) between the ZY and HY (Figure 2A). The taxonomic classification of differential metabolites in each fermentation stage is presented in Figure 2B. The main metabolite differences during fermentation between the HY and ZY occurred in fatty acids, carboxylic acids, and organic oxygen compounds. Microbes in fermentation can influence metabolite changes by interacting with each other. Xu et al. (2019) reported that *L. plantarum* can contribute to fatty acid oxidation during the processing of fermented fish paste, while Bian et al. (2022) found that carboxylic acids were positively related to *Lactobacillaceae* during Pu‐erh tea fermentation. In general, knowing about the function of the microbes during fermentation is conducive to the quality of YJS.

### 3.2 Analysis of N‐containing compounds

**FIGURE 3:**
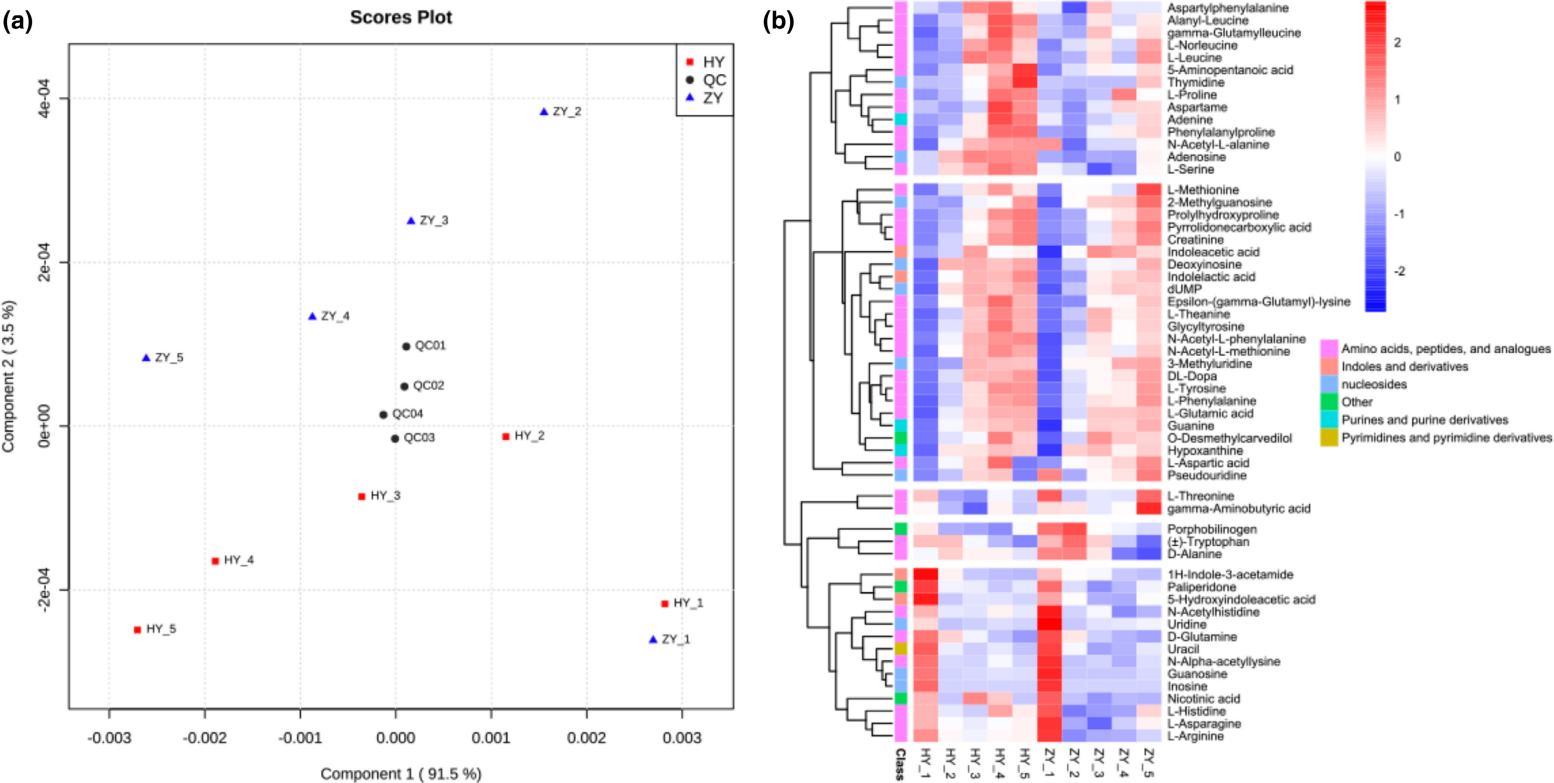
Scores plot of PLS‐DA model for N‐containing compounds (a). Heatmap for nitrogen‐containing organic compounds during YJS fermentation (b).

A total of 57 N‐containing compounds were identified, comprising 27 amino acids and derivatives, eight dipeptides, 10 nucleosides and nucleotides, four purines and pyrimidines, four indoles, and four other types of nitrogenous organics (including porphobilinogen, o‐desmethylcarvedilol, paliperidone, and nicotinic acid). Supervised partial least‐squares discrimination analysis (PLS‐DA) was used to analyze the differences in N‐containing compounds derived from different YJS groups (Szymańska et al., 2012). The results are shown in Figure 3a. According to the sampling time, the sample points were successively distributed in component 1 (variations: 91.5%), which indicated that the N‐containing compounds in YJS would gradually change with fermentation time. In addition, no obvious separation was found between HY‐1 and ZY‐1, and the difference in N‐containing compounds was minor in the first stage of fermentation. When HY was compared with ZY, the points were distributed along component 2 (variations: 3.5%), and the inoculation of strains had an impact on the change in nitrogen compounds during the fermentation process of YJS. As HY‐1/ZY‐1 was compared to other sample sites, HY‐1 was closer to other HY points, revealing that following the inoculation of *W. cibaria* and *L. plantarum*, the change range of N‐containing compounds was smaller than that of ZY, which may be attributable to the two strains' efforts to maintain microbial structures and consistently dominate during the fermentation process, thus reducing the fluctuation of N‐containing compounds.

According to the relative concentrations of the compounds in each sampling stage, K‐means clustering was used for N‐containing compound clustering in YJS. The results demonstrate that N‐containing compounds may be classified into five clusters based on their changing characteristics (Figure 3b). The N‐containing compounds in clusters I and II were generated throughout fermentation, with cluster I containing a higher final content in HY and cluster II possessing the opposite. The compounds in clusters IV and V were consumed by fermentation. The compounds in cluster III (L‐threonine and gamma‐aminobutyric acid) were detected with higher content in ZY eventually. The majority of N‐containing compounds accumulate during fermentation (clusters I and II). Some of these, such as L‐leucine, L‐proline, and L‐serine, contribute to the quality of fermented foods by adding flavor, taste, and nutrition (Maoz et al., 2022; Zhu et al., 2023). Furthermore, a number of N‐containing compounds, such as histidine (histamine), tyrosine (tyramine), and phenylalanine (phenylethylamine), had negative effects on YJS (Gao et al., 2023), but it was shown that the HY group controlled them with inoculation of *W. cibaria* and *L. plantarum*.

### 3.3 Correlation analysis of N‐containing compounds

The fermentation substrate is the most abundant source of small‐molecule N‐containing compounds in YJS. During fermentation, small‐molecule N‐containing compounds are constantly consumed and produced. Considering that, the correlation analysis was used to show the correlation between the N‐containing compounds in YJS under the two fermentation treatments.

**FIGURE 4:**
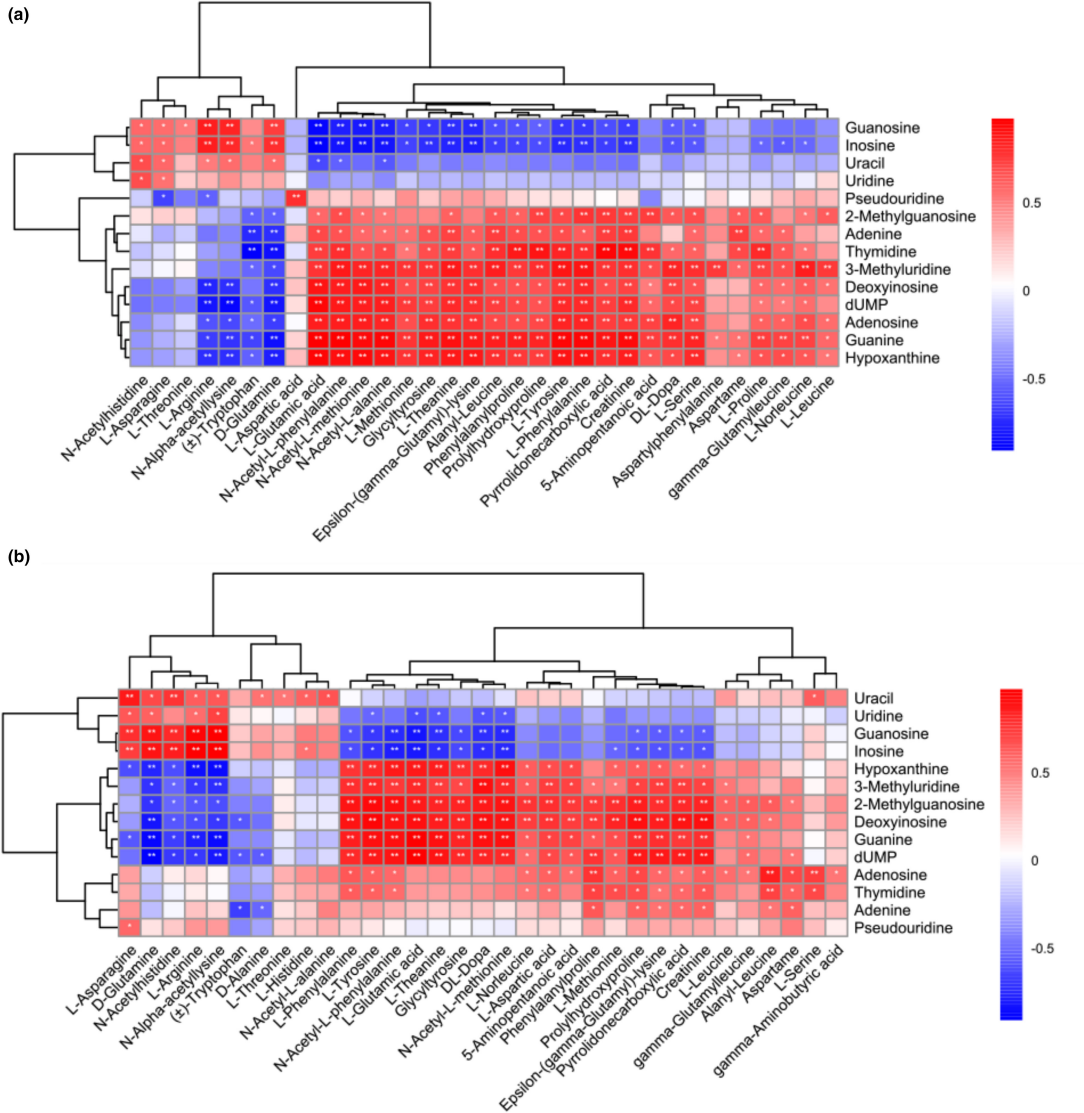
Correlation map of nucleic acid hydrolysates and amino acids (a, for HY; b, for ZY) (*p* < .01 marked with “**”, .01 < *p* < .05 marked with “*” in a and b)

Amino acids and peptides produced by protein hydrolysis and nucleosides, purines, and pyrimidines produced by nucleic acid hydrolysis were the types of N‐containing compounds, with the highest content in YJS. Hydrolysis led to a positive correlation between these substances, but negative correlations between some amino acid derivatives and some nucleic acid hydrolysates were shown in Figure 4. Besides, the same compounds were featured with different degrees of correlation between HY and ZY. In terms of guanosine and inosine, they were negatively correlated with most amino acids, but the correlation level of HY was higher than ZY. Apart from that, uracil was not positively related to most amino acids in ZY to a great extent, but was positively connected with seven amino acids in HY. In ZY, N‐acetyl histidine showed an extremely negative correlation with guanine, 2‐methylguanosine, hypoxanthine, deoxyinosine, dUMP, and 3‐methylluridine and a significantly positive correlation with guanosine, inosine, and uracil. In HY, N‐acetyl histidine had a positive correlation with guanosine, inosine, uracil, and uridine, while having an insignificantly negative correlation with other nucleotide hydrolysates.

The results indicated that some small N‐containing compounds in YJS may be more easily absorbed. The correlation coefficients between the relative concentrations of several N‐containing compounds were considerably different between HY and ZY. In addition to that, strong proteolysis was the main source of small‐molecule peptides and free amino acids in fermented products (Wang et al., 2017). Moreover, the inoculation of strains may affect the main change pathways of N‐containing compounds, including the hydrolysis of macromolecules and the decomposition and reuse of small molecules.

### 3.4 Metabolic pathway analysis

Small molecules of N‐containing compounds derived from raw materials were an important source of the taste and flavor of YJS and a source of nitrogen for microorganisms. The identified N‐containing compounds were mainly amino acids, peptides, purines, pyrimidines, and nucleotides. Degradation of hydrolysates and microbial reuse complicated the composition of N‐containing compounds in YJS. Here, it should be mentioned that most N‐containing compounds rapidly reached a relatively stable and high value in HY, and their relative concentration in the YJS is characterized by an increasing trend with time. Beyond that, the relative abundances of *Lactobacillus* and *Weissella* in fish–chili pastes increased rapidly within a week after adding commercial starters (Hua et al., 2021). Besides, in fermented production, a significant correlation between microorganisms and differential metabolites has been proved after inoculation of the autochthonous starter (Jiang et al., 2022; Randazzo et al., 2017). Based on this research, the differences in microbial community structure changes that resulted from the inoculation of the starter could be responsible for the differences in the relative concentrations of metabolites between HY and ZY.

**FIGURE 5:**
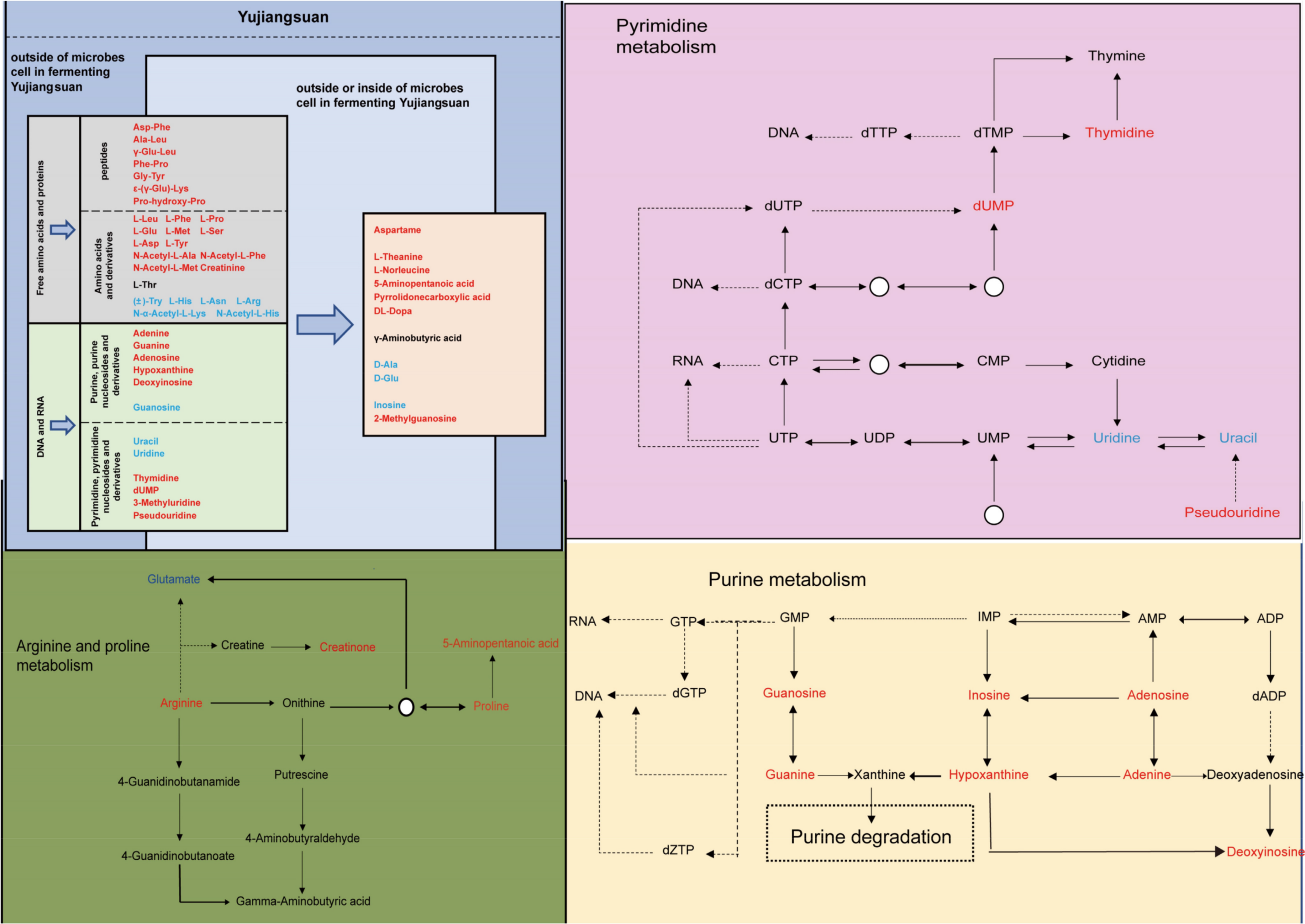
Schematic representation of the changes in small‐molecule N‐containing compounds; the increase in concentration of the compounds as the fermentation progresses is denoted in red, and vice versa in blue.

The metabolic pathways of the identified metabolites were analyzed by the KEGG database, which proved that *W. cibaria and L. plantarum* changed the content of N‐containing compounds in YJS by affecting the hydrolysis of macromolecular substances (Figure 5). Small N‐containing compounds, accumulated by protein and nucleic acid hydrolysis and consumed via decomposition, absorption, and utilization by microorganisms, experience complex dynamic change. N‐containing compounds can fall into two groups, namely amino acids and nucleic acid metabolites, depending on their metabolic source; the former impacts product quality by affecting taste, flavor, and nutrition, while the latter, including precursors and secondary metabolites of DNA and RNA, is a link in microbial growth.

A variety of metabolites were associated with nucleotide metabolism, such as purine metabolism and pyrimidine metabolism. These metabolites act as raw materials for the synthesis of DNA and RNA. After adenosine is converted into inosine or adenine, hypoxanthine can be further generated by the action of enzymes to enter the purine biodegradation pathway. In addition, inosine may participate in the purine salvage pathway. In this study, the concentrations of inosine and hypoxanthine were not significantly different between the two fermentation methods and exhibited a small change from day 4 to day 6 of fermentation. Therefore, degradation and microbial reuse are not the main causes of the difference in adenosine and adenine concentrations between the two fermentation treatments.

## 4 CONCLUSIONS

For the fermentation of YJS, the inoculation of *W. cibaria and L. plantarum* as starters led to metabolite changes in the fermentation process, and these changes mainly occurred in the middle stage of fermentation (4–12 days). Compared with traditional fermentation, the effect of inoculated fermentation was mainly reflected in the acceleration of the hydrolysis of biological macromolecules and the accumulation of small molecules, specifically N‐containing compounds. Furthermore, the hydrolysis efficiency of proteins and nucleic acids was improved, and the accumulation rate of amino acids, peptides, and nucleosides increased. In addition, the inoculation of *W. cibaria and L. plantarum* facilitated the rapid maturation of YJS, as the small molecule concentration reached a relatively stable state faster than traditional fermentation. The study of N‐containing compound dynamics will combine with physicochemical properties and sensory analysis to help the application of the starter in industrial production and contribute to the selection of functional strains. Besides, the inoculation of the autochthonous starter in fermentation can be a way to guarantee the quality of YJS.

## AUTHOR CONTRIBUTIONS

**Shirui Yu:** Writing – original draft (equal). **Wenkang Hu:** Investigation (equal). **Lina Ma:** Investigation (equal). **Yin Luo:** Resources (equal). **xuefeng zeng:** Project administration (equal). **Shanjun Tian:** Project administration (equal).

## FUNDING INFORMATION

This study was funded by the Science and Technology Project of Guizhou Province ([2019]2370), the Special Fund Project of the Central Government Guiding Local Science and Technology Development ([2019]4006), the Engineering Research Center of Higher Education in Guizhou Province (KY [2020]022), Moutai Institute High‐level Talents Research Initiation Project (mygccrc[2022]087), and Moutai Institute Characteristic Food Resources Comprehensive Utilization Talent Base.

## CONFLICT OF INTEREST STATEMENT

The authors declare no conflict of interest.

## Data Availability

The data that support the findings of this study are available on request from the corresponding author.

## References

[fsn33674-bib-0001] Bekhit, A. E. A. , Holman, B. W. B. , Giteru, S. G. , & Hopkins, D. L. (2021). Trends in Food Science & Technology Total volatile basic nitrogen (TVB‐N) and its role in meat spoilage: A review. Trends in Food Science & Technology, 109, 280–302.

[fsn33674-bib-0002] Bian, X. , Miao, W. , Zhao, M. , Zhao, Y. , Xiao, Y. , Li, N. , & Wu, J. L. (2022). Microbiota drive insoluble polysaccharides utilization via microbiome‐metabolome interplay during Pu‐erh tea fermentation. Food Chemistry, 377, 132007. 34999465 10.1016/j.foodchem.2021.132007

[fsn33674-bib-0003] Chen, S. , Fu, Y. , Bian, X. , Zhao, M. , Zuo, Y. , Ge, Y. , Xiao, Y. , Xiao, J. , Li, N. , & Wu, J. L. (2022). Investigation and dynamic profiling of oligopeptides, free amino acids and derivatives during Pu‐erh tea fermentation by ultra‐high performance liquid chromatography tandem mass spectrometry. Food Chemistry, 371, 131176. 34601212 10.1016/j.foodchem.2021.131176

[fsn33674-bib-0004] Dan, T. , Hu, H. , Li, T. , Dai, A. , He, B. , & Wang, Y. (2022). Screening of mixed‐species starter cultures for increasing flavour during fermentation of milk. International Dairy Journal, 135, 105473.

[fsn33674-bib-0005] Gao, X. , Li, C. , He, R. , Zhang, Y. , Wang, B. , Zhang, Z.‐H. , & Ho, C.‐T. (2023). Research advances on biogenic amines in traditional fermented foods: Emphasis on formation mechanism, detection and control methods. Food Chemistry, 405, 134911.

[fsn33674-bib-0006] Hu, W. , Liu, Z. , Fu, B. , Zhang, X. , Qi, Y. , Hu, Y. , Wang, C. , Li, D. , & Xu, N. (2022). Metabolites of the Soy Sauce Koji making with *Aspergillus niger* and *Aspergillus oryzae* . International Journal of Food Science and Technology, 57, 301–309.

[fsn33674-bib-0007] Hu, W. , Yang, X. , Ji, Y. , & Guan, Y. (2021). Effect of starter cultures mixed with different autochthonous lactic acid bacteria on microbial, metabolome and sensory properties of Chinese northeast sauerkraut. Food Research International, 148, 110605. 34507749 10.1016/j.foodres.2021.110605

[fsn33674-bib-0008] Hua, Q. , Gao, P. , Xu, Y. , Xia, W. , Sun, Y. , & Jiang, Q. (2020). Effect of commercial starter cultures on the quality characteristics of fermented fish‐chili paste. LWT, 122, 109016.

[fsn33674-bib-0009] Hua, Q. , Sun, Y. , Xu, Y. , Gao, P. , & Xia, W. (2021). Bacterial community succession and biogenic amine changes during fermentation of fish‐chili paste inoculated with different commercial starter cultures. International Journal of Food Science & Technology, 56, 6752–6764.

[fsn33674-bib-0010] Hua, Q. , Sun, Y. , Xu, Y. , Gao, P. , & Xia, W. (2022). Contribution of mixed commercial starter cultures to the quality improvement of fish‐chili paste, a Chinese traditional fermented condiment. Food Bioscience, 46, 101559.

[fsn33674-bib-0011] Jiang, L. , Liu, L. , Chen, H. , Zhang, W. , He, L. , & Zeng, X. (2022). Effects of autochthonous starter cultures on bacterial communities and metabolites during fermentation of Yu Jiangsuan, a Chinese traditional fermented condiment. LWT, 168, 113874.

[fsn33674-bib-0012] Lai, T. , Shuai, L. , Han, D. , Lai, Z. , Du, X. , Guo, X. , Hu, W. , Wu, Z. , & Luo, T. (2021). Comparative metabolomics reveals differences in primary and secondary metabolites between “Shixia” and “Chuliang” longan (*Dimocarpus longan* Lour.) pulp. Food Science & Nutrition, 9, 5785–5799. 34646546 10.1002/fsn3.2552 PMC8498058

[fsn33674-bib-0013] Liu, L. , Wu, J. , Yang, J. , Tang, Z. , & Zeng, X. (2020). Isolation and fermentation characteristics of γ‐aminobutyric acid‐producing lactic acid bacteria from Yujiangsuan, a traditional Miao ethnic fermented condiment. Food Science, 42(18), 73–79. (In Chinese, with English abstract). 10.7506/spkx1002-6630-20200914-182

[fsn33674-bib-0014] Maoz, I. , Lewinsohn, E. , & Gonda, I. (2022). Amino acids metabolism as a source for aroma volatiles biosynthesis. Current Opinion in Plant Biology, 67, 102221. 35533493 10.1016/j.pbi.2022.102221

[fsn33674-bib-0015] Randazzo, C. L. , Todaro, A. , Pino, A. , Pitino, I. , Corona, O. , & Caggia, C. (2017). Microbiota and metabolome during controlled and spontaneous fermentation of Nocellara Etnea table olives. Food Microbiology, 65, 136–148. 28399996 10.1016/j.fm.2017.01.022

[fsn33674-bib-0016] Saccenti, E. , Hoefsloot, H. C. J. , Smilde, A. K. , Westerhuis, J. A. , & Hendriks, M. M. W. B. (2014). Reflections on univariate and multivariate analysis of metabolomics data. Metabolomics, 10, 361–374.

[fsn33674-bib-0017] Szymańska, E. , Saccenti, E. , Smilde, A. K. , & Westerhuis, J. A. (2012). Double‐check: Validation of diagnostic statistics for PLS‐DA models in metabolomics studies. Metabolomics, 8, 3–16. 22593721 10.1007/s11306-011-0330-3 PMC3337399

[fsn33674-bib-0018] Wang, W. , Xia, W. , Gao, P. , Xu, Y. , & Jiang, Q. (2017). Proteolysis during fermentation of Suanyu as a traditional fermented fish product of China. International Journal of Food Properties, 20, S166–S176.

[fsn33674-bib-0019] Xu, Y. , Li, L. , Xia, W. , Zang, J. , & Gao, P. (2019). The role of microbes in free fatty acids release and oxidation in fermented fish paste. LWT, 101, 323–330.

[fsn33674-bib-0020] Yang, K. , Wu, K. , Zhang, J. , Gu, Y. , Liu, G. , & Guan, C. (2021). Analysis of amino acid content in fish sauce acid seasoning. China Condiment, 46(6), 144‐148. (In Chinese, with English abstract). 10.3969/j.issn.1000-9973.2021.06.029

[fsn33674-bib-0021] Yao, H. , Yang, J. , Zhan, J. , Lu, Q. , Su, M. , & Jiang, Y. (2021). Preparation, amino acid composition, and in vitro antioxidant activity of okra seed meal protein hydrolysates. Food Science & Nutrition, 9, 3059–3070. 34136171 10.1002/fsn3.2263 PMC8194734

[fsn33674-bib-0022] Zheng, Y. , Yang, P. , Chen, E. , Song, H. , Li, P. , Li, K. , & Xiong, J. (2020). Investigating characteristics and possible origins of off‐odor substances in various yeast extract products. Journal of Food Biochemistry, 44, 1–9. 10.1111/jfbc.13184 32163601

[fsn33674-bib-0023] Zhu, Y. , Liu, J. , & Liu, Y. (2023). Understanding the relationship between umami taste sensitivity and genetics, food‐related behavior, and nutrition. Current Opinion in Food Science, 50, 100980.

[fsn33674-bib-0024] Zotta, T. , Di Renzo, T. , Sorrentino, A. , Reale, A. , & Boscaino, F. (2022). Selection of non‐saccharomyces wine yeasts for the production of leavened doughs. Microorganisms, 10, 1–13. 10.3390/microorganisms10091849 PMC9501029 36144451

